# Adverse events in the postoperative period of cardiac surgery in a pediatric intensive care unit: the contribution of the VIS score and the RACHS-1

**DOI:** 10.5935/2965-2774.20230215-en

**Published:** 2023

**Authors:** Ana Beatriz Ramos Wasniewski, Claudia Pires Ricachinevsky, Raíssa Queiroz Rezende, Bruna Tomasi Lorentz, Edinara da Silva Silveira, Viviane Helena Rampon Angeli, Mariana González de Oliveira, Themis Reverbel da Silveira

**Affiliations:** 1 Pediatric Intensive Care Unit, Hospital da Criança Santo Antônio, Irmandade Santa Casa de Misericórdia de Porto Alegre - Porto Alegre (RS), Brazil; 2 Instituto do Coração, Hospital de Clínicas, Faculdade de Medicina, Universidade de São Paulo - São Paulo (SP), Brazil; 3 Universidade Federal de Ciências da Saúde de Porto Alegre - Porto Alegre (RS), Brazil

**Keywords:** Patient safety, Safety management, Postoperative period, Cardiac surgical procedures/adverse effects, Cardiovascular agents, Intensive care units, pediatric

## Abstract

**Objective:**

To evaluate the occurrence of adverse events in the postoperative period of cardiac surgery in a pediatric intensive care unit and to find any patient characteristics that can predict such events.

**Methods:**

This was a historical cohort study of patients recovering in the pediatric intensive care unit for the first 7 days after cardiac surgery between April and December 2019, by reviewing the medical records. The following were reviewed: demographic, clinical, and laboratory characteristics; patient severity scores; and selected adverse events, grouped into device-related, surgical, and nonsurgical.

**Results:**

A total of 238 medical records were included. At least one adverse event occurred in 110 postoperative patients (46.2%). The total number of adverse events was 193 (81%). Vascular catheters were the most common cause, followed by cardiac arrest, bleeding, and surgical reexploration. In the univariate analysis, the vasoactive-inotropic score (VIS), Risk Adjustment in Congenital Heart Surgery (RACHS-1) score, age, Pediatric Index of Mortality (PIM-2), cardiopulmonary bypass and aortic clamping duration were significantly associated with adverse events. In the multivariate analysis, VIS ≥ 20 (OR 2.90; p = 0.004) and RACHS-1 ≥ 3 (OR 2.11; p = 0.019) were significant predictors, while age and delayed sternal closure showed only trends toward significance. To predict the occurrence of adverse events from VIS and RACHS-1, the area under the curve was 0.73 (95%CI 0.66 - 0.79).

**Conclusion:**

Adverse events were quite frequent in children after cardiac surgery, especially those related to devices. The VIS and RACHS-1, used together, predicted the occurrence of adverse events well in this pediatric sample.

## INTRODUCTION

Children undergoing cardiac surgery are at high risk of morbidity and mortality. Factors in the immediate postoperative period that may indicate their shortand long-term outcomes should be identified and quantified.^(1)^ The mortality rate of patients undergoing cardiac surgery varies between institutions and reflects the potential to improve quality of care by identifying and mitigating risk factors associated with worse outcomes.^(2)^

Much remains to be learned about adverse events (AEs) in children with heart disease.^(3)^ These patients often undergo cardiac procedures with a high risk for systematic errors, making them dependent on a highly specialized team.^(4)^ They are more vulnerable to events because they have a limited physiological capacity to tolerate them.^(3)^ It is noteworthy that in pediatric cardiac surgery, AEs usually occur in a chain, with different apex situations according to the location of the patient: in the operating room, for example, AEs are likely related to cardiopulmonary bypass, whereas in the intensive care unit (ICU) they will be related to extubation and reduction of vasoactive drugs, among others.^(5)^

The prediction of complications in the cardiac postoperative period is the first and critical step toward improving patient safety and outcomes. There is no specific tool to predict the occurrence of complications of pediatric cardiac surgery.^(6)^ In the cardiac surgery scenario, effective communication, with standardized methods and technical training, both for the surgical team and the intensive care team, are crucial. It is important that a program of improvements for patient safety be implemented.^(3)^

The objective of the present study was to evaluate the occurrence of AEs in the postoperative period of cardiac surgery in a pediatric ICU and to find any patient characteristics that could predict AEs.

## METHODS

This was a historical cohort study of the first 7 postoperative days of patients undergoing cardiac surgery at the pediatric ICU of *Hospital da Criança Santo Antônio* in Porto Alegre, Rio Grande do Sul, Brazil, with a mean of 125 hospitalizations and 35 heart surgeries per month. Its pediatric ICU is a quaternary care unit with patients with different types of complex pathologies, cardiac postoperative periods, transplants, and various congenital malformations. It also delivers advanced clinical care for nonsurgical diseases. The unit has 40 beds for patients up to 18 years of age. The hospital is linked to the *Universidade Federal de Ciências da Saúde de Porto Alegre* with a Medical Residency Program in Pediatrics and Pediatric Intensive Care.

Data were collected through remote access to electronic medical records by the principal investigator and research assistants. The first 7 postoperative days in the pediatric ICU were reviewed. To preserve the uniformity of collection, all data were reviewed by the main researcher.

The sample was defined by convenience and by the inclusion and exclusion criteria described below. The study included medical records of postoperative patients from cardiac surgery performed between April and December 2019 who were admitted to the pediatric ICU, aged 0 to 18 years, of both sexes, with a minimum stay of 2 hours in the pediatric ICU. We excluded medical records that could not be retrieved despite an active search by various means, those treated at a location other than the pediatric ICU after surgery, and medical records with missing clinical or laboratory data that made it impossible to calculate the scores needed for the study.

The characterization of the sample included age in months, sex, weight in kilograms, diagnosis of congenital defects of the cardiovascular system,^(7)^ Risk Adjustment in Congenital Heart Surgery score (RACHS-1),^(8)^ Pediatric Index of Mortality (PIM-2),^(9)^ and vasoactive-inotropic score (VIS)^(10)^ in the immediate postoperative period, as well as the presence and duration of cardiopulmonary bypass (CPB), aortic clamping, and delayed sternal closure.

The AEs studied were grouped into three categories: surgical (cardiac tamponade; surgical reexploration; bleeding); nonsurgical (pneumothorax or hemothorax; cardiac arrest; pressure injury); and device-related (accidental extubation; complications with vascular catheters; complications with catheter bladder or chest tube). These AEs were chosen because they are considered to have relevant clinical-care repercussions and are less influenced by the vagaries of medical records.

The AE severity classification was performed according to the World Health Organization (WHO):

- Mild: mild symptoms, loss of function or minimal-to-moderate damage, rapid duration, only minimal interventions needed.
- Moderate: symptomatic symptoms requiring intervention.
- Severe: symptomatic symptoms requiring intervention for life support or major clinical/surgical intervention, causing a decrease in life expectancy, great damage, or permanent or long-term loss of function.
- Death: shortly after surgery, the event caused or accelerated death.^(11)^

The outcome of the patients was classified as discharge from the pediatric ICU, death, and others (readmission to the pediatric ICU and transfer to another service).

The study was approved by the Research Ethics Committee of the *Irmandade Santa Casa de Misericórdia de Porto Alegre* (ISCMPA), with opinion 3,963,919, and was performed according to the ethical standards established in the Declaration of Helsinki of 1964 and its subsequent amendments or equivalent ethical standards. It was registered on the Brazil Platform under CAAE 27472619.7.0000.5683. The informed consent form and the assent form were not needed because this was an observational study that did not interfere with patient care.

Quantitative data are described as the mean and standard deviation. To break the Gaussian assumptions, we used the median and interquartile range (P25 - P75). Categorical data are expressed as counts and percentages. Comparisons between quantitative variables were performed using Student’s *t* test or its nonparametric substitute (Mann-Whitney test). In situations of categorical variables, we used the chi-squared test or, when necessary, Fisher’s exact test.

To evaluate the association between selected variables and the occurrence of AEs, we used a logistic regression model. This model estimated effect magnitudes and the probability of occurrence of events. This method yielded odds ratios and their 95% confidence intervals (95%CI) through univariate and then multivariate models with mutually adjusted estimates. From the probability of AEs estimated by the model, we obtained its predictive performance, which is expressed as the area under the receiver operating characteristic (ROC) curve. Findings with p ≤ 0.05 were considered statistically significant. The analyses were performed with IBM’s Statistical Package for the Social Sciences (SPSS), version 27.0.

## RESULTS

Of the 261 postoperative cardiac records, 238 met the study inclusion criteria. The other 23 records were excluded because two were not located, seven patients died in the operating room, five recovered outside the pediatric ICU, and nine had missing data for the calculation of scores. The characteristics of the population are shown in table 1.

**Table 1:** Sociodemographic, clinical, and laboratory characteristics and outcomes of patients undergoing cardiac surgery

Variables	
Age at surgery (months)	6.7 (1.7 - 25.4)
Sex	
Male	130 (54.6)
Female	108 (45.4)
Weight (kg)	5.6 (3.3 - 11.9)
Diagnosis	
Venous return abnormalities	10 (4.2)
VA connection anomalies	24 (10.1)
Septal defects	75 (31.5)
Heart abnormalities R	65 (27.3)
Heart abnormalities L	17 (7.1)
Abnormalities of the thoracic arteries	37 (15.6)
Miscellaneous	4 (1.7)
Not applicable	6 (2.5)
RACHS-1	
1	33 (13.9)
2	85 (35.7)
3	82 (34.5)
4	19 (8)
6	13 (5.4)
Not applicable	6 (2.5)
PIM-2	3.1 (2.1 - 5.1)
VIS	27.6 (12.5 - 37.8)
CPB	192 (80.7)
CPB time (minutes)	104 (69.8 - 140.8)
Aortic clamping	169 (71.0)
Time of aortic clamping (minutes)	65 (41 - 101.5)
Delayed sternal closure	31 (13.0)
Time for sternum closure (days)	2 (1 - 3)
Length of stay (days)	9 (3 - 22)
Outcome	
Discharge from the pediatric ICU	196 (82.3)
Death	38 (16.0)
Others	4 (1.7)

VA - ventricle-arterial; R - right; L - left; RACHS-1 - Risk Adjustment in Congenital Heart Surgery; PIM-2 - Pediatric Index of Mortality; VIS - vasoactive-inotropic score; CPB - cardiopulmonary bypass; ICU - intensive care unit. Values are expressed as median and interquartile range (P25-P75) or n (%).

The AEs surveyed are detailed in table 2. The total number of AEs was 193 (81%). The most frequent category was device-related. Of them, complications with vascular catheters were the most common. Nonsurgical AEs were the second most frequent, and surgical AEs were the least.

**Table 2:** Adverse events investigated in the cardiac postoperative records

Total number of adverse events	
Related to invasive devices	89 (46.1)
Complications with vascular catheters	66 (34.2)
Complications with urinary catheter or chest tube	15 (7.8)
Accidental extubation	8 (4.1)
Related to surgical aspects	49 (25.4)
Bleeding	25 (13.0)
Surgical reexploration	22 (11.4)
Cardiac tamponade	2 (1.0)
Related to nonsurgical aspects	55 (28.5)
Cardiac arrest	36 (18.7)
Pneumothorax or hemothorax	11 (5.7)
Pressure injury	8 (4.1)

Values expressed as n (%).

In 110 postoperative patients (46.2%), at least one AE occurred. The characterization of the two groups of patients - with and without AEs - is shown in table 3. Lower age and weight, higher PIM-2 score, higher VIS, higher RACHS-1 score (3 - 4), delayed sternal closure, longer CPB and aortic clamping were significantly associated with AEs. The RACHS-1 score 1 was significantly associated with the absence of AEs. Regarding outcomes, patients with AEs were more likely to die, and patients without AEs were more likely to be discharged from the pediatric ICU.

**Table 3:** Associations of the study variables of interest with adverse events in the cardiac postoperative period

Variables	No adverse event (n = 128)	With adverse event (n = 110)	p value
Age at surgery (months)	8 (2.7 - 72.5)	5.7 (0.5 - 9.8)	0.003^*^
Sex			0.679†
Male	72 (56.3)	58 (52.7)	
Female	56 (43.7)	52 (47.3)	
Weight (kg)	6.7 (3.8 - 18.8)	4.9 (3.1 - 7.7)	0.00^*^
RACHS-1			< 0.001†
1	27 (21.8)‡	6 (5.6)	
2	52 (41.9)	33 (30.6)	
3	35 (28.2)	47 (43.5)‡	
4	6 (4.9)	13 (12.0)‡	
6	4 (3.2)	9 (8.3)	
PIM-2	2.6 (1.7 - 4.1)	3.7 (2.7 - 7.0)	< 0.001^*^
VIS	20 (5 - 37.5)	36.8 (26.6 - 47.5)	< 0.001^*^
CPB	103 (80.5)	89 (80.9)	1.000†
CPB time (minutes)	88 (62 - 123)	117 (94.5 - 160.5)	< 0.001^*^
Aortic clamping	89 (69.5)	80 (72.7)	0.690†
Median aortic clamping time (minutes) (P25-P75)	55 (31.5 - 89)	72.5 (51.3 - 112.8)	0.003^*^
Delayed sternal closure	7 (5.5)	24 (21.8)	< 0.001†
Time to sternal closure (days)	2 (2 - 3)	1 (1 - 3)	0.139^*^
Length of stay (days)	5 (3 - 14)	14 (7 - 28)	< 0.001^*^
Outcome			< 0.001†
Discharge from the pediatric ICU	120 (93.8)‡	76 (69.1)	
Death	5 (3.9)	33 (30.0)‡	
Others	3 (2.3)	1 (0.9)	

RACHS-1 - Risk Adjustment in Congenital Heart Surgery; PIM-2 - Pediatric Index of Mortality; VIS - vasoactive-inotropic score; CPB - cardiopulmonary bypass; ICU - intensive care unit.

* Mann-Whitney test; † chi-squared test; ‡ statistically significant association by the residue test adjusted to 5% significance level. Values are expressed as median and interquartile range (P25-P75) or n (%).

**Figure 1:**
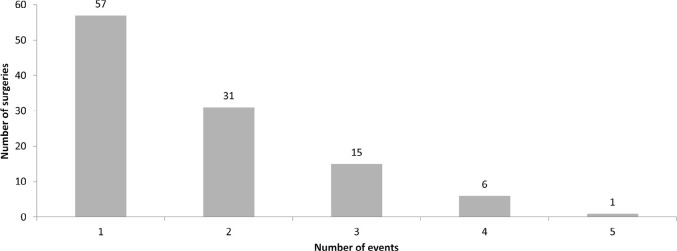
Adverse events during surgery.

Figure 1 shows the distribution of the total number of AEs in the postoperative period evaluated. There was one AE in 57 postoperative patients (51.8%), two in 31 patients (28.2%), three in 15 patients (13.6%), four in six patients (5.5%), and five in one patient (0.9%).

The association between selected variables and the occurrence of AEs, as calculated by the logistic regression model, is shown in table 4. In the univariate analysis, VIS ≥ 20, RACHS-1 ≥ 3, age ≤ 24 months, PIM-2 ≥ 5, duration of CPB ≥ 120 minutes, and aortic clamping time ≥ 30 minutes had a positive relationship with the occurrence of AEs. When performing multivariate analysis, VIS ≥ 20 (OR 2.90; p = 0.004), and RACHS-1 ≥ 3 (OR 2.11; p = 0.019) were still statistically significant, while age and delayed sternal closure had only a tendency toward an association.

**Table 4:** Logistic regression of variables for the occurrence of adverse events

Characteristic	Univariate analysis			Multivariate analysis		
	OR	95%CI	p value	OR	95%CI	p value
VIS ≥ 20	4.65	2.52 - 8.59	< 0.001	2.90	1.40 - 6.04	0.004
RACHS-1 ≥ 3	3.11	1.82 - 5.31	< 0.001	2.11	1.13 - 3.95	0.019
Age ≤ 24 months	2.97	1.58 - 5.58	0.001	1.89	0.92 - 3.85	0.082
PIM-2 ≥ 5	2.54	1.39 - 4.65	0.002	0.98	0.47 - 2.04	0.957
CPB time ≥ 120 minutes	1.25	1.03 - 1.50	0.021	0.88	0.68 - 1.14	0.350
Aortic clamping time ≥ 30 minutes	1.78	1.04 - 3.02	0.034	1.12	0.57 - 2.20	0.740
Sex	1.15	0.70 - 1.90	0.590	1.10	0.62 - 1.95	0.740
Delayed sternal closure	4.82	1.99 - 11.70	0.001	2.64	0.89 - 7.83	0.080

OR - odds ratio; 95% CI - 95% confidence interval; VIS - vasoactive-inotropic score; RACHS-1 - Risk Adjustment in Congenital Heart Surgery; PIM-2 - Pediatric Index of Mortality; CPB - cardiopulmonary bypass.

**Figure 2:**
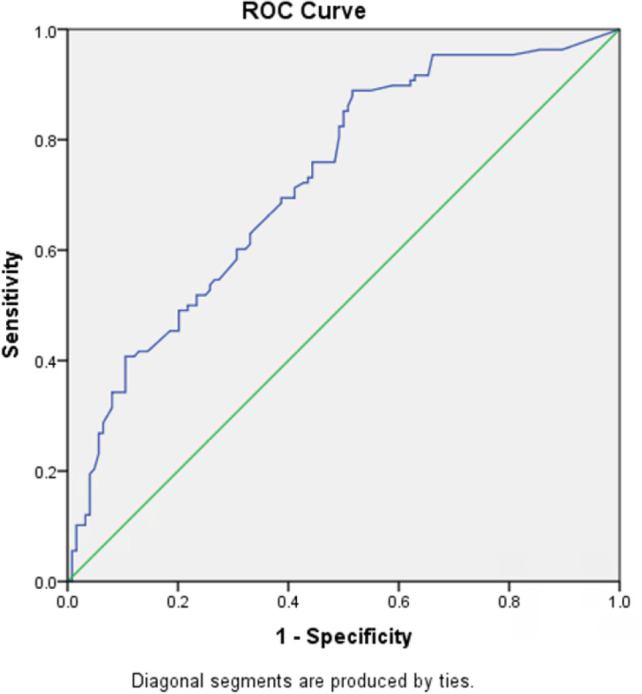
Area under the ROC curve for the prediction of adverse events based on the vasoactive-inotropic score and the *Risk Adjustment in Congenital Heart Surgery*.

Figure 2 shows that considering the predicting the occurrence of AEs from only VIS and RACHS-1, the area under the ROC curve was 0.73 (95%CI 0.66 - 0.79).

Area under the curve = 0.73 (95% confidence interval 0.66 - 0.79).

Of all the AEs, 50.3% were classified as mild, followed by 37.3% as severe. The AEs related to devices were mostly mild (84.3%). Half of those related to surgical aspects were classified as severe (51%). Those related to nonsurgical aspects were mostly (60%) of severe intensity, and cardiac arrest was the one most often directly associated with death (25%). The classification of the severity of the AEs is shown in Table 5.

**Table 5:** Adverse events and damage intensity classification

Adverse events	Total	Damage intensity classification			
		Mild	Moderate	Severe	Death
Related to invasive devices	89 (46.1)	75 (84.3)	0 (0.0)	14 (15.7)	0 (0.0)
Complication with vascular catheters	66 (34.2)	66 (100)	0 (0.0)	0 (0.0)	0 (0.0)
Complication with urinary catheter or chest tube	15 (7.8)	9 (60.0)	0 (0.0)	6 (40.0)	0 (0.0)
Accidental extubation	8 (4.1)	0 (0.0)	0 (0.0)	8 (100)	0 (0.0)
Related to surgical aspects	49 (25.4)	11 (22.5)	12 (24.5)	25 (51)	1 (2.0)
Bleeding	25 (13.0)	11 (44.0)	0 (0.0)	13 (52.0)	1 (4.0)
Surgical reexploration	22 (11.4)	0 (0.0)	12 (54.5)	10 (45.5)	0 (0.0)
Cardiac tamponade	2 (1.0)	0 (0.0)	0 (0.0)	2 (100)	0 (0.0)
Related to nonsurgical aspects	55 (28.5)	11 (20)	2 (3.6)	33 (60)	9 (16.4)
Cardiac arrest	36 (18.7)	0 (0.0)	0 (0.0)	27 (75.0)	9 (25.0)
Pneumothorax or hemothorax	11 (5.7)	3 (27.3)	2 (18.2)	6 (54.5)	0 (0.0)
Pressure injury	8 (4.1)	8 (100)	0 (0.0)	0 (0.0)	0 (0.0)
Total	193 (100)	97 (50.3)	14 (7.2)	72 (37.3)	10 (5.2)

Results expressed as n (%).

## DISCUSSION

The occurrence of AEs clearly contributes to worse outcomes of patients in the cardiac postoperative period. Knowing in advance which patients are at greater risk of AEs is essential for their clinical management and better postoperative evolution. As we do not have a specific tool for this purpose,^(6)^ the present study sought factors that could predict the occurrence of selected AEs.

The incidence of AEs in children in health services ranges from 1 to 62%.^(12)^ Studies using the trigger detection method in pediatric ICUs have found an AE rate as high as 76%.^(13)^ Regarding the number of patients affected, 59-62% of those admitted to the pediatric ICU will have suffered at least one AE.^(14,15)^ In studies of children undergoing cardiac surgery, 32-43% had at least one event.^(16,17)^ Our numbers are close to those reported in the literature. We found a higher overall incidence, but similar in the number of patients affected in the cardiac postoperative period.

The AE in the present study were most often related to devices, especially to vascular catheters. These data are in line with the literature. Loss of a peripherally inserted central catheter was the most frequent AE in one Brazilian study, at n = 18 (25%).^(18)^ Another study from Brazil showed events related to vascular access as the most prevalent, with 227 events (40.8%).^(19)^ In an international publication, events associated with equipment such as lines and tubes had the second highest rate, with 101 events (22%).^(20)^ In the present study, the second most frequent events were related to nonsurgical aspects, especially cardiac arrest. In a multicenter study, the total cardiopulmonary resuscitation rate was 2.6%, a lower figure than ours.^(21)^ Another rate lower than ours - the occurrence of cardiopulmonary resuscitation in 20 of the 325 patients evaluated (6.1%) - was indicated in a retrospective study of a single center.^(17)^ A cardiopulmonary resuscitation rate of 52%, which was higher than we found, was obtained in a study that analyzed deaths in pediatric cardiac surgery programs.^(22)^ As for the order of frequency of bleeding, our findings are within the range reported in the literature. Rates of 7% of patients with bleeding,^(17)^ smaller than ours, and a total frequency of bleeding of 35%,^(22)^ which is higher than that obtained here, have been found in the literature. Regarding surgical reexploration, data similar to ours were reported in a study in which 11% of newborns needed early cardiac surgical reintervention.^(23)^ Lower numbers than those in the present study were reported, at 3.5%,^(24)^ 5.6%,^(25)^ and 5.5% reintervention.^(17)^ One study reported that 32% of their patients needed unscheduled surgical reintervention.^(22)^

Regarding the intensity of damage, about half of the events were classified as mild, a finding that differs from the literature.^(15,26)^ The majority of mild events were related to devices. Cardiac arrest and surgical reexploration, which have an established relationship with higher risk of death,^(17,21,23-25)^ were often of greater severity in the present study.

Age, weight, time of CPB, time of aortic cross-clamping, delayed sternal closure, and PIM-2 were relevant variables in the univariate analysis. Data similar to these have been reported in the literature: children who underwent cardiac surgery with postoperative complications were younger, had lower weight, and were shorter.^(6)^ A positive association with complications has also been reported for surgical time, longer CPB and aortic clamping times, and delayed sternal closure.^(6)^ Another study showed that children younger than 1 year of age had a higher rate of complications.^(16)^ A longer CPB time was a risk factor for a higher occurrence of cardiac and noncardiac complications.^(17)^ PIM-2, when used as a trigger for AE prediction, yielded easy and fast identification of patients at risk of AEs.^(27)^

In general, regardless of age or severity at the time of admission to the pediatric ICU, the occurrence of AEs is associated with worse outcomes, including higher morbidity and mortality rates.^(20,28)^ This finding is confirmed in the present study, as death was relevant in the group of patients with AE.

Given the lack of a specific tool to predict the occurrence of AEs in the postoperative period of pediatric cardiac surgery and analyzing the possible associations of the selected variables with AEs, we found the important contribution of the VIS and the RACHS-1. They have been used to predict morbidity, mortality, and length of hospital stay. In the present study, their combined use was good at predicting AEs in the postoperative period of pediatric cardiac surgery.

The VIS is one of the variables that can influence negative outcomes in the cardiac postoperative period. In children, high VIS predicts unfavorable outcomes, including morbidity and mortality after cardiac surgery.^(29)^ Children with high doses of vasoactive drugs in the immediate postoperative period have a high probability of morbidity and mortality.^(30)^ VIS at 2 hours postoperatively was identified as an independent factor for predicting low output syndrome.^(31)^ The occurrence of low output syndrome was associated with a higher number of complications.^(31)^ Elevated VIS at 48 hours postoperatively was considered an alert that the patient was at risk of unfavorable outcomes.^(32)^ In another study, a higher VIS was associated with mortality at 30 days after surgery, cardiac arrest, need for dialysis, neurological disorders, duration of mechanical ventilation, and length of stay in the pediatric ICU.^(10)^ Another study showed that the maximum VIS at 48 hours after surgery was associated with unfavorable outcomes, morbidity, and mortality.^(1)^ The same study reported that although a higher VIS could be a marker of poor cardiac physiology in the immediate period after surgery, it may lead to more prolonged therapies, complications, and poor cardiopulmonary recovery.^(1)^

In the literature, we did not find data on any specific relationship between VIS and AEs, which, despite making it difficult to compare our results, adds some novelty to our study. We believe that we have demonstrated the importance of VIS as a predictor of the occurrence of AEs. Based on our literature review, we defined a high VIS as ≥ 20 in the immediate postoperative period.

The RACHS-1 score is widely used to predict the mortality of patients in the cardiac postoperative period. We also found studies on its use for other outcomes. As a death prediction model, RACHS-1 showed moderate discriminatory power, with an area under the ROC curve of 0.68 (95%CI 0.58 - 0.79).^(33)^ It also showed good power of discrimination between survivors and nonsurvivors in a publication by our center, with an area under the ROC curve of 0.70 (95%CI 0.63 - 0.77).^(34)^ For the prediction of death, Brazilian studies showed good predictive power.^(35)^

For postoperative cardiac complications in children, RACHS-1 had good predictive power (area under the ROC curve of 0.68) in a recent study.^(6)^ Higher RACHS-1 has been associated with the occurrence of AEs (p < 0.001) and has been a good predictor of them.^(36,37)^ Patients with higher RACHS-1 were more likely to have complications in the cardiac postoperative period.^(16,17)^ The present study corroborates the finding that higher RACHS-1 scores were associated with AEs. Specifically, RACHS-1 scores of 3 and 4 were associated with the occurrence of AEs.

In the present study, the combined use of VIS and RACHS-1 had good discriminatory power for the occurrence of AEs in children after cardiac surgery, with an AUC of 0.73. The higher the VIS or RACHS-1 in the immediate postoperative period, the more likely an AE was. We believe that this finding may be applicable in the care and prevention of AEs in these patients.

Among the limitations of the present study, its external validity may be low because it was performed in a single pediatric ICU. The duration of the study and the detection of AEs, performed by reviewing medical records, may have influenced the identification of events.

On the other hand, as a strong point of the present study, the identification of the combined use of VIS and RACHS-1 in the specific prediction of AEs in the postoperative period of cardiac surgery in children may be of value in clinical practice.

## CONCLUSION

Adverse events were frequent in the pediatric population undergoing cardiac surgery. The most frequent adverse events were complications from devices. The vasoactive-inotropic score and the Risk Adjustment in Congenital Heart Surgery could be considered adequate elements to predict the occurrence of adverse events in the pediatric cardiac postoperative period.
